# Control of the committed step in lipopolysaccharide biosynthesis

**DOI:** 10.1016/j.jbc.2026.113238

**Published:** 2026-06-09

**Authors:** Wei Mi, Rajkanwar Nathawat, Hongpeng Wang, Sofia Delgado

**Affiliations:** 1 Department of Pharmacology, Yale University School of Medicine, New Haven, Connecticut, USA; 2 Department of Molecular Biophysics and Biochemistry, Yale University, New Haven, Connecticut, USA; 3 Department of Microbial Pathogenesis, Yale University School of Medicine, New Haven, Connecticut, USA; 4 Department of Cell Biology, Yale University School of Medicine, New Haven, Connecticut, USA

**Keywords:** lipopolysaccharide (LPS) biogenesis, LpxC, regulated proteolysis, coordination of LPS, phospholipid, peptidoglycan synthesis, deacetylase activity, allosteric regulation, FtsH, LapB, YejM

## Abstract

Lipopolysaccharide (LPS) is an essential component of the outer membrane of most Gram-negative bacteria. It maintains envelope integrity and forms a permeability barrier that protects cells from environmental stress, host defenses, and many antibiotics. LPS biosynthesis must be tightly controlled to ensure proper outer membrane assembly while preventing the toxic accumulation of intermediates. Central to this control is LpxC, a deacetylase that catalyzes the committed step in LPS biosynthesis and directs metabolic flux into the pathway. In *Escherichia coli*, LpxC abundance is primarily controlled by proteolysis mediated by the membrane-bound AAA + protease FtsH. This process is regulated by two essential membrane proteins, LapB and YejM. LapB acts as an adaptor that promotes LpxC degradation, whereas YejM functions as an antiadaptor that counteracts this activity. Through this regulatory network, signals from multiple stages of LPS biogenesis, along with inputs from other cell envelope biosynthetic pathways, are integrated to maintain LPS homeostasis. In addition to proteolytic control, new evidence suggests that LpxC enzymatic activity can also be modulated. Here, we summarize current understanding of the mechanisms governing LpxC regulation in *E. coli* and highlight unresolved questions. Further elucidation of these regulatory mechanisms may provide new opportunities for antibiotic development.

Gram-negative bacteria possess a cell envelope composed of two membranes: the cytoplasmic or inner membrane (IM) and the outer membrane (OM). Between these membranes lies the periplasmic space, which contains a thin layer of peptidoglycan (1) (Fig. 1*A*). The OM is an asymmetric lipid bilayer, with lipopolysaccharide (LPS) in the outer leaflet and phospholipids in the periplasmic-facing inner leaflet (2). LPS is an essential glycolipid for most Gram-negative bacteria and consists of three structural regions: lipid A, core oligosaccharide, and O-antigen (3) (Fig. 1*B*). The OM functions as a selective permeability barrier, restricting the entry of hydrophobic compounds and large hydrophilic molecules. This barrier property is largely attributable to LPS, which plays a central role in maintaining the selective permeability and mechanical stability of the cell envelope (2, 4, 5).

**Figure 1:**
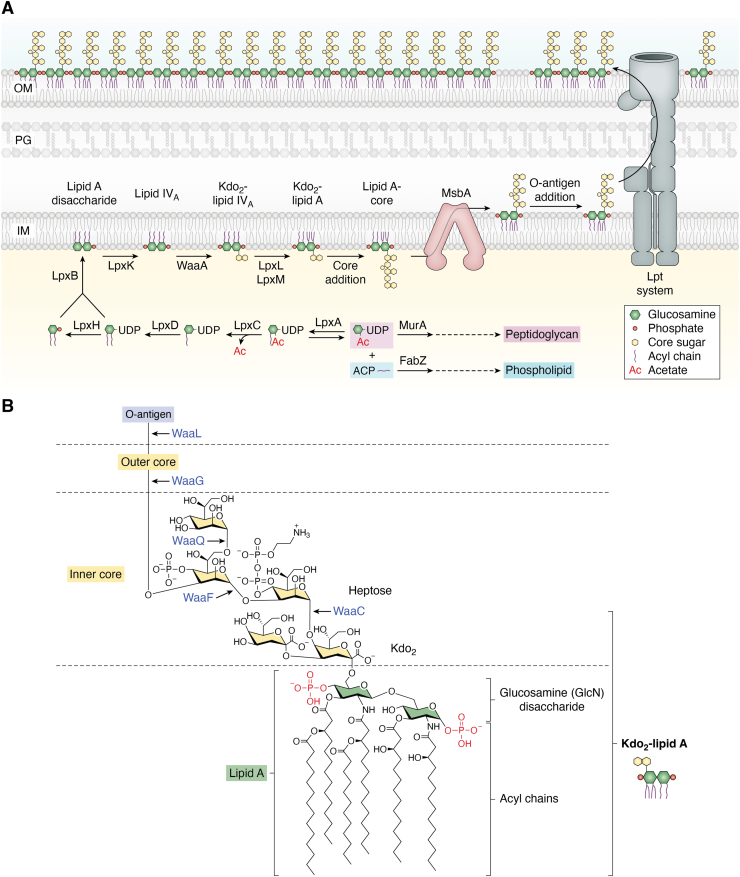
**LPS synthesis and transport.***A*, LPS synthesis begins in the cytoplasm with reversible acylation of UDP *N*-acetylglucosamine (UDP-GlcNAc) using *R*-3-hydroxymyristol-ACP, precursors shared with the peptidoglycan (PG) and phospholipid biosynthesis, respectively. The product of LpxA catalyzed reaction is then irreversibly deacetylated by LpxC, an enzyme which determines the rate of LPS synthesis. Following multiple enzymatic reactions, Kdo_2_-lipid A is generated, which represents the minimal essential LPS molecule. Core oligosaccharide sugars are then added by respective glycosyl transferases to form the lipid A-core which is flipped to the periplasmic leaflet of the inner membrane (IM) by flippase, MsbA. To the flipped lipid A-core, O-antigen is added by enzyme, WaaL. This complete LPS molecule is then transported to outer membrane (OM) by LPS transport (Lpt) system. *B*, chemical structure of conserved lipid A-inner core from *E. coli*, with the enzymes responsible for adding inner core sugars to Kdo_2_-lipid A. ACP, acyl carrier protein; LPS, lipopolysaccharide.

LPS synthesis begins in the cytoplasm and proceeds at the cytoplasmic leaflet of the IM with the formation of the lipid A-core precursor. The lipid A biosynthetic pathway is highly conserved among Gram-negative bacteria and has been most extensively characterized in *Escherichia coli* (3, 6) (Fig. 1). It starts with the LpxA-catalyzed transfer of an acyl chain from *R*-3-hydroxymyristoyl-acyl carrier protein (ACP) to uridine-5′-diphosphate-acetyl-D-glucosamine (UDP-GlcNAc) (7, 8). These substrates are also shared with other essential envelope biosynthetic pathways: *R*-3-hydroxymyristoyl-ACP is an intermediate in phospholipid synthesis, whereas UDP-GlcNAc is a precursor for peptidoglycan biosynthesis. Because LpxA-catalyzed reaction is reversible and thermodynamically unfavorable (9), flux through the pathway is primarily determined by the second enzyme in the pathway, LpxC.

LpxC is a deacetylase that catalyzes the removal of the acetyl group from UDP-3-O-acyl-GlcNAc (10) (Fig. 2*A*). Structurally, LpxC consists of two homologous domains connected by a linker. Despite limited sequence conservation, the two domains adopt similar fold composed of a β-sheet and two α-helices. The domains pack together so that the α-helices are sandwiched between the two β-sheets. Each domain also contains a characteristic subdomain, termed as insertion, that is unique to LpxC enzymes (11, 12). Insertion I, located in domain I, consists of a small antiparallel β-sheet formed by three β-strands, whereas insertion II in domain II adopts a β-α-β structural motif (Fig. 2*B*). A catalytic zinc ion resides at the interface of the two domains, forming the active site, which is solvent-exposed (Fig. 2*C*). Adjacent to the catalytic center, a hydrophobic tunnel, formed by insertion II, accommodates the acyl chain of the substrate and contributes to substrate specificity (13). Because this deacetylation reaction is irreversible, LpxC effectively controls the partitioning of the shared precursors *R*-3-hydroxymyristoyl-ACP and UDP-GlcNAc among LPS, phospholipid, and peptidoglycan biosynthesis, thereby establishing LpxC as the key enzyme in envelope homeostasis.

**Figure 2:**
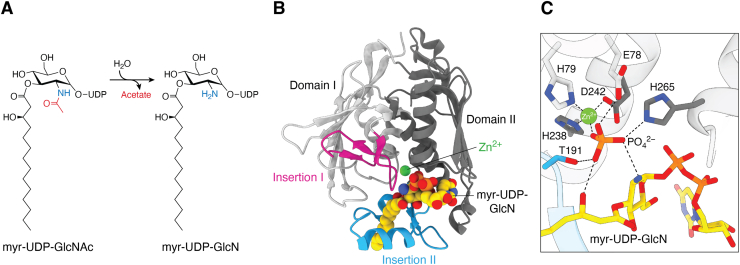
**Structure and active site of *E. coli* LpxC.***A*, LpxC-catalyzed deacetylation of myr-UDP-GlcNAc. *B*, crystal structure of LpxC (PDB ID: 4MDT) showing two domains (domain I in *light gray* and domain II in *dark gray*) and the insertions associated with each domain (insertion I in *pink* and insertion II in *blue*). The reaction product, myr-UDP-GlcN, is shown as *yellow spheres*, positioned at the interface between the domains. The acyl chain of the product is accommodated in the hydrophobic passage formed by the insertion II helix. The catalytic Zn^2+^ ion (*green sphere*) is situated at the interface of the two domains. *C*, close-up view of the active site highlighting coordination of the Zn^2+^ ion with residues H79, H238, and D242, and the bound phosphate (mimic of LpxC reaction byproduct—acetate ion). Additional residues critical for catalysis, including E78, T191, and H265, are highlighted. The deacetylated product, myr-UDP-GlcN is shown in stick representation (*yellow*). PDB, Protein Data Bank; UDP-GlcNAc, uridine-5′'-diphosphate-acetyl-D-glucosamine.

Subsequent steps of LPS synthesis are catalyzed by LpxD (14), LpxH (15), and LpxB (8, 16), generating a lipid A disaccharide intermediate. LpxK then phosphorylates the 4′-hydroxyl group of the distal glucosamine to produce lipid IV_A_, a tetra-acylated disaccharide 1,4′-bisphosphate (17). The 3-deoxy-D-manno-octulosonic acid (KDO) transferase WaaA (also known as KdtA) adds KDO residues to lipid IV_A_ (18), and the late acyltransferases LpxL and LpxM incorporate secondary acyl chains to form KDO_2_-lipid A (19, 20). KDO_2_-lipid A represents the minimal LPS structure required for viability under most conditions in *E. coli*, whereas lipid IV_A_ alone supports growth only in specific genetic backgrounds (21). Additional sugars are subsequently added to assemble the complete lipid A-core structure on the cytoplasmic side of the IM. The core oligosaccharide comprises inner and outer regions assembled by glycosyltransferases encoded by the *gmhD* and *waaQ* operons, with the inner core being more conserved and essential for OM integrity, and the outer core more variable (3). The ABC transporter MsbA flips the lipid A-core to the periplasmic leaflet of the IM (22, 23), where the O-antigen is ligated by WaaL (24). The fully assembled LPS molecule is then transported across the periplasm and inserted into the OM *via* the LPS transport pathway, a complex of seven proteins that forms a transenvelope bridge facilitating LPS movement and insertion (25, 26).

Both insufficient and excessive production of LPS are lethal, underscoring the need for tight control of LPS synthesis (27, 28, 29). Insufficient LPS synthesis and/or compromised LPS transport to the OM, impair OM integrity and weaken the permeability barrier, whereas excessive LPS production leads to the toxic accumulation of LPS in the IM (30, 31). LPS synthesis and transport are coordinated through multiple regulatory nodes, as detailed in several recent reviews (32, 33, 34). Among these mechanisms, LpxC occupies a central position: as the committed step enzyme, it determines pathway flux and is the primary regulatory node. In this article, we focus on the mechanisms governing LpxC regulation in *E. coli*, highlighting current models, unresolved discrepancies, and outstanding questions.

## A dynamic proteolytic switch: the FtsH/LapB/YejM pathway in LpxC control

Following the discovery that LpxC catalyzes the committed step in LPS biosynthesis, Raetz *et al*. showed that conditions leading to reduction of lipid A synthesis, such as partial loss-of-function mutations in *lpxA* or *lpxD,* resulted in a marked increase in LpxC deacetylase activity in *E. coli,* without obvious changes in the activities of the other five enzymes in the lipid A synthesis pathway (9, 14, 35). These findings provided strong evidence for feedback control in LPS biosynthesis and established LpxC as a principal regulatory node governing pathway flux. Kinetic analysis revealed that the increase in LpxC activity was primarily due to an elevated *V*_max_, with little change in *K*_M_ (35). The enhanced enzymatic activity was attributable to increased LpxC protein levels rather than changes in catalytic efficiency. The elevated LpxC abundance did not arise from increased *lpxC* transcription, indicating that posttranscriptional regulation—particularly protein degradation—plays a key role in controlling LpxC abundance. However, the protease responsible for LpxC degradation was not identified in these early studies (35).

**Figure 3:**
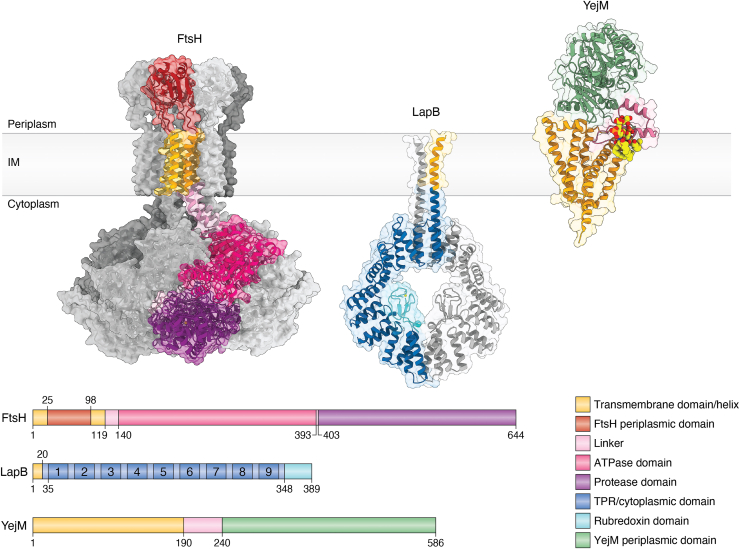
**Structure and domain organization of FtsH, LapB, and YejM.** Structures of FtsH hexamer (AlphaFold3 model), LapB dimer (AlphaFold3 model), and YejM (crystal structure: PDB ID: 6XLP) are shown in cartoon and surface representation. FtsH and LapB are rendered in *gray* to emphasize the overall oligomeric architecture, while one protomer is highlighted and colored according to their domain organization. For FtsH, the periplasmic domain (*red*), transmembrane helices (*gold*), ATPase domain (*deep pink*), and protease domain (*purple*) are indicated. For LapB, the transmembrane helix (*gold*), TPR (tetratricopeptide repeat)/cytoplasmic domain (*dark blue*), and rubredoxin domain (*light blue*) are shown. For YejM, the transmembrane domain (*gold*), linker (*light pink*), and periplasmic domain (*sage green*) are highlighted, with bound LPS molecule depicted as *yellow spheres*. Horizontal lines indicate the approximate boundaries of the periplasm, inner membrane (IM), and cytoplasm. Domain architectures and residue boundaries are summarized in the schematic below. LPS, lipopolysaccharide; PDB, Protein Data Bank.

A breakthrough came in 1999, when Ogura *et al*. demonstrated that a temperature-sensitive mutant of *ftsH*, which encodes an essential membrane-bound AAA + protease, caused accumulation of LpxC at the nonpermissive temperature (31). FtsH is the only essential AAA + protease in *E*. *coli* and degrades a wide range of protein substrates (36). It consists of an N-terminal transmembrane (TM)/periplasmic domain, a central ATPase domain, and a C-terminal protease domain (Fig. 3). FtsH assembles into a hexamer in which the ATPase domains harness the energy of ATP hydrolysis to unfold protein substrates. These unfolded polypeptides are then threaded through the central pore of the hexamer into a concealed chamber formed by the C-terminal protease domains, where they are degraded. In the same study, Ogura *et al*. further showed that purified FtsH directly degrades LpxC *in vitro,* establishing FtsH as the primary protease responsible for LpxC turnover and, consequently, for determining its cellular abundance. Recently, two additional essential membrane proteins, LapB (originally named YciM) and YejM (named PbgA in *Salmonella*) were identified as key regulators of LpxC degradation (32, 37, 38). The essentiality of the *ftsH*, *lapB*, and *yejM* genes in *E. coli* underscores the critical importance of tightly controlling LpxC abundance to maintain LPS homeostasis.

LapB contains a single N-terminal TM helix, nine cytoplasmic tetratricopeptide repeats, and a C-terminal rubredoxin domain (39) (Fig. 3). LapB forms a homodimer with a planar architecture (40). In 2014, the Reddy laboratory identified *lapB* (*yciM*) as an essential gene and proposed that LapB functions as an adaptor protein by specifically recruiting LpxC to FtsH for degradation (41). Work from the Raina laboratory independently supported LapB’s role in accelerating LpxC degradation. They also isolated a *lapB* deletion mutant which could survive only under specific conditions and exhibited severe growth defects along with pleiotropic defects in LPS synthesis, leading to the designation of LapB as LPS assembly protein B (42).

YejM is a membrane protein composed of five N-terminal TM helices, a linker, and a large periplasmic domain (43) (Fig. 3). Genetic analyses indicate that YejM regulates LPS synthesis. The *E. coli* LH530 strain, which carries a premature stop codon in *yejM* just downstream of the TM region, produces a truncated YejM variant and synthesizes reduced amounts of LPS (44, 45). Consistent with an essential role for YejM, complete deletion of the *yejM* gene is lethal (43). YejM (PbgA) in *Salmonella* was initially proposed to function as a cardiolipin transporter (46), but subsequent genetic analyses revised this interpretation and instead implicated YejM as a regulator of LPS synthesis (47). In 2020, four groups independently reported that suppressor mutations rescuing *yejM* truncations or depletion repeatedly mapped to *ftsH*, *lapB*, or *lpxC*, converging on the conclusion that loss of YejM function reduces cellular LpxC amounts, causing cell death in *E. coli* (30, 48, 49, 50). Among these studies, the Silhavy laboratory demonstrated that YejM stabilizes LpxC by modulating its degradation through the FtsH/LapB machinery. They further showed that *yejM* is no longer essential in a *lapB* deletion background (constructed in strains carrying *lpxC101* with reduced deacetylase activity), suggesting that YejM acts upstream of LapB (30). The Bernhardt laboratory independently demonstrated that YejM antagonizes LpxC degradation and physically interacts with LapB, further supporting the model that YejM regulates FtsH/LapB-mediated LpxC proteolysis (48).

Biochemical and structural analysis provided more mechanistic insights. Early interpretations of YejM crystal structures were guided by its proposed role as a cardiolipin transporter (51, 52), and consequently misassigned the bound lipid density as cardiolipin (51). In 2020, Rutherford *et al*. reported the *E. coli* YejM/LPS structure, demonstrating that the previously assigned “cardiolipin” density actually corresponds to LPS (53). The structure revealed that YejM’s periplasmic linker region interacts with GlcN(I)-1-phosphate, the reducing-end-derived glucosamine of lipid A (53). These findings suggest a model in which YejM senses LPS accumulation in the periplasmic leaflet of the IM. Peptides derived from this linker region bind LPS specifically, supporting this sensing model (53). In parallel, a reconstituted proteoliposome system containing purified FtsH, LapB, and YejM was used to measure *in vitro* LpxC degradation kinetics in a membrane lipid environment (54). These studies showed that LapB functions as an adaptor, enhancing LpxC degradation by increasing its affinity for FtsH. Further biochemical analysis revealed that the TM helix of LapB is required for interaction with FtsH, while its cytoplasmic domain recruits LpxC (54). A cryo-EM structure of the LapB_cyto_/LpxC complex demonstrated that a LapB dimer recruits two LpxC molecules (55). In addition, the YejM/LapB complex structure revealed that two YejM molecules sandwich the LapB TM helices, consistent with an antiadaptor role in which YejM sequesters LapB away from FtsH (54). Structural superimposition of YejM/LPS and YejM/LapB complexes revealed that LPS and LapB bind overlapping sites on YejM, indicating that they compete for YejM binding (54).

**Figure 4:**
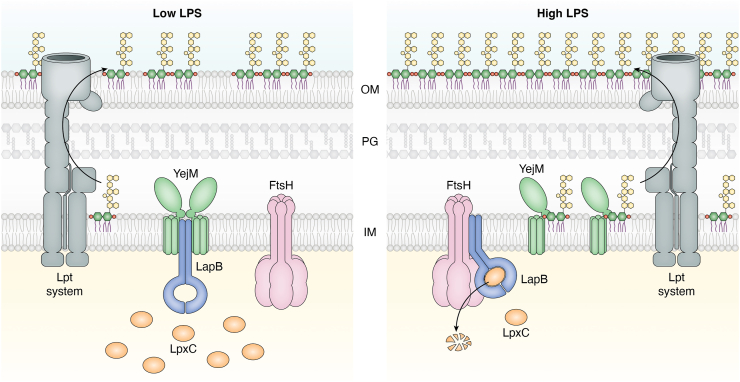
**Model for LPS homeostasis in *E. coli*.** Under low LPS conditions, newly synthesized LPS molecules are continuously transported from inner membrane (IM) to outer membrane (OM) by LPS transport (Lpt) system, leading to low concentration of LPS in the IM. In this condition, YejM associates with LapB and sequesters its transmembrane helices, preventing interaction with the protease FtsH. Consequently, the LPS biosynthetic enzyme LpxC is stabilized due to the absence of FtsH/LapB-mediated degradation, and LPS synthesis continues. When LPS amount is more than required in the OM, LPS accumulated in the periplasmic leaflet of IM binds to YejM and releases LapB. LapB can then engage with FtsH, promoting the degradation of LpxC and downregulating LPS biosynthesis. LPS, lipopolysaccharide; PG, peptidoglycan.

Together, these biochemical and structural findings led us to propose a model of LpxC degradation regulated by cellular LPS levels in *E. coli* (54) (Fig. 4). When LPS amounts are low, YejM sequesters LapB, limiting LpxC degradation by FtsH and maintaining high LpxC abundance to promote LPS synthesis. When LPS accumulates in the periplasmic leaflet of the IM, LPS binding to YejM disrupts the YejM/LapB complex. The liberated LapB then associates with FtsH, stimulates LpxC degradation, and lowers its abundance, thereby reducing LPS synthesis. This model provides a mechanistic explanation for feedback control of LPS levels through modulation of LpxC abundance. Because the model is primarily derived from *in vitro* and structural studies, further *in vivo* analyses are needed to test it, as some reports suggest that YejM and LapB form a complex constitutively (53, 56).

## Beyond a simple antiadaptor/adaptor framework: expanded roles of YejM and LapB in LPS homeostasis

The identification of YejM and LapB as key regulators of LpxC degradation represented major progress in the field. However, accumulating evidence suggests that both YejM and LapB have additional regulatory functions beyond their roles as antiadaptor and adaptor, respectively. These findings highlight the complexity of LPS synthesis regulation.

In the current model, YejM functions as an antiadaptor by engaging LapB through its TM helices (48, 54). However, deletion of YejM’s periplasmic domain reduces LpxC and LPS amounts in *E. coli* (30, 44, 49), even though this domain is not involved in LapB binding. These observations suggest that YejM’s periplasmic domain plays an additional, yet undefined, role in regulating LPS synthesis. Some genetic findings further challenge the notion that YejM functions solely as an antiadaptor. The Misra group showed that point mutations in LapB (L95R or A126V) rescue YejM truncation; notably, LapB protein is not detectable in these mutants, effectively mimicking a *lapB* null allele (49). Similarly, the Raina group reported that a truncated YejM (*lapC377fs*) suppresses the lethality associated with deletion of *lapB* (50). These genetic relationships are difficult to reconcile with a model in which YejM functions only through physical interaction with LapB. Together, these data suggest that YejM acts beyond merely modulating LapB availability, likely involving additional functions of its periplasmic domain.

The periplasmic domain of YejM shares structural similarity with members of the hydrolase superfamily, including EptA, which modifies lipid A with phosphoethanolamine, and LtaS, a lipoteichoic acid synthase found in Gram-positive bacteria (57). A magnesium-binding site has been identified in the periplasmic domain of YejM, which exhibits phosphatase activity. Interestingly, although the putative active site resides in the periplasmic domain, only full-length YejM displays magnesium-dependent phosphatase activity, whereas the isolated periplasmic domain shows markedly reduced activity (57). The physiological substrate of YejM remains unknown. The observation that only full-length YejM exhibits phosphatase activity suggests that its substrate may reside in the membrane, possibly a lipid substrate. It has also been proposed that YejM’s periplasmic domain might regulate LPS synthesis through direct interaction with FtsH (49); however, *in vivo* data do not support a stable YejM-FtsH interaction (48, 53). Defining the function of the YejM periplasmic domain, such as identifying its potential substrate and binding partners, may provide insight into its broader role in LPS homeostasis.

The adaptor function of LapB in regulating LPS synthesis in *E. coli* is well established now; however, there are studies which indicate additional roles of LapB in maintaining LPS homeostasis. The *lapB* deletion mutant of *E. coli* isolated by the Raina group, produced elevated levels of LPS enriched with heterogeneous lipid A-core species, including penta-acylated lipid A and incompletely assembled core structures, suggesting that LapB may have functions beyond serving as an adaptor for FtsH (42). One possible explanation is that LpxC accumulation in a Δ*lapB* background increases flux into lipid A synthesis and overwhelms downstream enzymes. However, suppressor mutations of Δ*lapB* were mapped to the *waaC* gene and *waaQ* operon, which encode glycosyltransferases involved in core oligosaccharide assembly (42) (Fig. 1). These findings indicate that LapB coordinates lipid A synthesis with core oligosaccharide assembly, thereby coupling the biosynthesis of these two regions of LPS.

LapB contains a C-terminal rubredoxin domain, which is typically associated with electron transfer (39, 40). Structural analysis of the LapB_cyto_/LpxC complex shows that the rubredoxin domain lies adjacent to the LpxC substrate-binding site, which led to the discovery that LapB directly inhibits LpxC deacetylase activity (55). The proximity of the rubredoxin domain to LpxC raises the possibility that cytoplasmic redox status modulates LpxC activity and thereby couples cellular redox state to LPS synthesis. Such a mechanism could provide a means for bacterial cells to coordinate LPS biogenesis with metabolic or respiratory states that alter cellular redox balance. Exploring how redox cues might influence LapB function may therefore reveal new principles linking envelope biogenesis to cellular physiology.

Finally, recent structural studies investigating the interaction between FtsH and IM protein complex HflKC, which plays an important role in protein quality control and cellular stress responses, have shown that HflKC encloses FtsH in a cage-like structure (58, 59, 60). Thus, even after LapB recruits LpxC, access to FtsH for proteolysis may itself be subject to additional regulation. Collectively, these structural, genetic, and biochemical observations argue that LPS synthesis regulation by the FtsH/LapB/YejM pathway involves multiple regulatory layers that extend well beyond a simple antiadaptor/adaptor paradigm.

## Multisite surveillance: spatial control of LPS synthesis and transport

**Figure 5:**
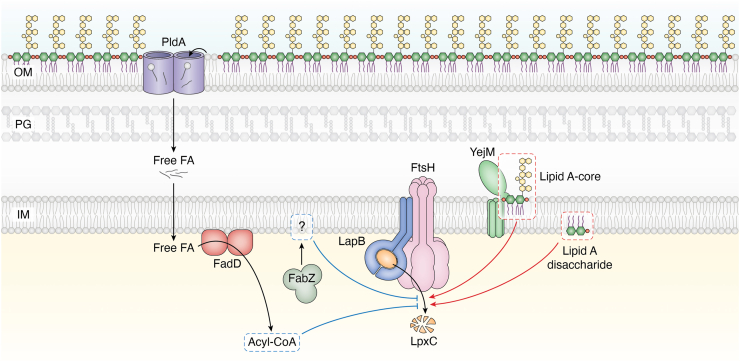
**Multisite surveillance system for LpxC degradation control.** FtsH/LapB/YejM-mediated degradation of LpxC is regulated by multiple signals, originated during LPS and phospholipid biosynthesis at different cellular sites. Activating signals are shown in *red* and the inhibiting signals are shown in *blue*. See main text for details. FA, fatty acid; IM, inner membrane; LPS, lipopolysaccharide; OM, outer membrane; PG, peptidoglycan.

As LPS molecules traverse three distinct cellular locations—synthesized at the cytoplasmic leaflet of the IM, flipped to the periplasmic leaflet, and then transported directly to the outer leaflet of the OM—*E. coli* monitors LPS status at multiple sites and employs distinct mechanisms to sense LPS at each location (Fig. 5). At the cytoplasmic leaflet of the IM, early lipid A intermediates provide feedback signals that regulate LpxC degradation. At the periplasmic leaflet of the IM, where LPS concentration is low, YejM detects even small fluctuations in LPS abundance. In contrast, the OM contains a large reservoir of LPS in its outer leaflet, making direct sensing of LPS levels difficult; therefore, *E. coli* monitors OM integrity indirectly by detecting phospholipid mislocalization. Signals originating from these locations ultimately converge to modulate LpxC abundance and adjust LPS synthesis.

At the cytoplasmic leaflet of the IM, lipid A-core is synthesized. The initial evidence for metabolite-dependent feedback was reported in the same study that established LpxC as the committed-step enzyme of lipid A synthesis (35). Inhibition of the first three enzymes in the pathway led to LpxC accumulation, whereas inhibition of KdsB, the CMP-KDO synthetase required for KDO production, caused lipid IV_A_ accumulation without altering LpxC abundance (35). These results suggest that early lipid A intermediates, rather than downstream products such as lipid IV_A_, mediate feedback regulation of LpxC degradation. Emiola *et al.* developed a quantitative model incorporating the nine enzymes involved in lipid A synthesis and proposed that lipid A disaccharide, produced by LpxB and consumed by LpxK, likely serves as a signaling molecule (61). Consistent with this model, overexpression of LpxK stabilizes LpxC, presumably because increased LpxK activity lowers intracellular levels of lipid A disaccharide (61).

Once fully assembled, lipid A-core is flipped to the periplasmic leaflet of the IM. At this stage, LPS is rapidly transferred to the OM (62), keeping its steady-state concentration low in the periplasmic leaflet of the IM. Consequently, even modest fluctuations in LPS concentration at this location can be detected more readily. The LPS level at this location can also serve as a sensitive indicator to coordinate LPS synthesis with downstream transport, preventing the accumulation of LPS intermediates when export is limiting. In the current model of regulated LpxC degradation (Fig. 4), YejM senses LPS accumulation at the periplasmic leaflet of the IM and modulates LpxC degradation (53, 54).

At the OM, LPS forms the major component of the outer leaflet, creating a large and relatively stable reservoir that may limit direct sensing of small fluctuations in LPS abundance. Instead, *E. coli* monitors OM integrity by detecting phospholipid mislocalization. The Silhavy group identified a dominant *mlaA*∗ mutant (*mlaA* encodes a component of the Mla system that normally prevents phospholipids from entering the outer leaflet), which permits phospholipids to enter the outer leaflet, disrupting OM asymmetry and leading to increased LpxC abundance in the cytoplasm (63). Further genetic analyses indicated that LpxC accumulation is dependent on PldA, an OM phospholipase that hydrolyzes mislocalized phospholipids and generates lysophospholipids and free fatty acids. These fatty acids are transported back into the cytoplasm and activated by FadD to form acyl-CoA, which in turn promotes LpxC accumulation (64). The Misra lab also reported that deletion of *pldA* in a *yejM* truncation background (*yejM*1163) caused a growth defect (49), supporting a genetic interaction between *pldA* and *yejM*. Recently, lysophospholipids, the other products of PldA-mediated phospholipid hydrolysis, were found to suppress phospholipid synthesis, providing a complementary mechanism to rebalance LPS and phospholipid biosynthesis (65).

Signals originating from the OM and the cytoplasmic leaflet of the IM are assumed to converge on the FtsH/LapB/YejM regulatory system (41, 63, 64), although direct experimental validation remains limited. If all regulatory inputs indeed converge on this system, an important question is how chemically distinct potential signaling molecules, such as lipid A disaccharide and acyl-CoA, are detected and used to regulate LpxC degradation. As the FtsH/LapB/YejM system spans the cytoplasm, IM, and periplasm, it is well positioned to integrate signals from multiple compartments. Nevertheless, additional unidentified proteins that detect signals and relay them to this regulatory module cannot be excluded.

When multiple signals from different locations regulate LpxC degradation, another critical question arises: how are conflicting inputs integrated? For example, inhibition of KDO synthesis leads to accumulation of the LPS intermediate lipid IV_A_, while fully assembled LPS amounts decrease; yet, LpxC abundance remains unchanged, contrary to the expectation that LpxC amount would rise to restore fully assembled LPS (35). Similarly, depletion of LptD disrupts OM lipid asymmetry and would be expected to elevate LpxC abundance to stimulate LPS synthesis, as observed in the *mlaA*∗ background. Instead, LpxC amounts decrease, likely because LPS accumulates in the periplasmic leaflet of the IM, triggering enhanced LpxC turnover (53). These observations indicate the presence of a signaling hierarchy. Signals originating from the cytoplasmic leaflet appear dominant, followed by those from the periplasmic leaflet of the IM, whereas OM-derived signals may be subordinate. This hierarchy may allow the cell to prevent accumulation of potentially toxic intermediates before restoring OM barrier integrity. Elucidating how these spatially distributed signals are integrated remains a central challenge in understanding LpxC regulation and LPS homeostasis.

## Metabolic coupling: balancing LPS and phospholipid synthesis

Maintenance of OM lipid asymmetry requires tight coordination between LPS and phospholipid synthesis. Both pathways draw from a common pool of a fatty acid precursor, *R*-3-hydroxymyristoyl-ACP, which can either be incorporated directly into LPS or further elongated to long-chain acyl-ACPs (*e.g.*, palmitoyl-ACP) for phospholipid production (9, 31) (Fig. 1). At steady state, the cellular LPS-to-phospholipid ratio is approximately 0.12 (7), which corresponds to roughly one quarter of synthesized fatty acids being incorporated into LPS. How *E. coli* achieves this balanced distribution of acyl flux has been a longstanding question in the field.

Genetic and biochemical studies have established coordination between FabZ and LpxC as a major mechanism coupling phospholipid and LPS synthesis (31). FabZ, an essential β-hydroxyacyl-ACP dehydratase in the fatty acid biosynthesis pathway, competes with LpxA for *R*-3-hydroxymyristoyl-ACP (66). Consistent with functional coupling, *fabZ* resides in an operon with lipid A biosynthetic genes (*lpxD, lpxA,* and *lpxB*) (66). FabZ was originally identified by the Raetz group. They reported that point mutations in *fabZ* that reduce dehydratase activity suppress the cell growth defect at 42 °C in strains carrying the temperature-sensitive *lpxA*2 allele (66). They further demonstrated that reduced FabZ activity also suppresses *lpxC* mutations (*e.g.*, *envA*1) that compromise LpxC deacetylase function (66). The Misra laboratory observed similar results while studying OM protein assembly, as they identified *fabZ* mutations that revert the phenotypes of hypomorphic *lpxC* alleles (*e.g.*, *asmB1*) (67). Subsequently, Ogura *et al*. reported that gain-of-function *fabZ* mutations (*e.g.*, *sfhC21*) suppress the lethality of *ftsH* temperature-sensitive mutants, which accumulate excess LpxC and overproduce LPS (31). Supporting these findings, the Zhou laboratory showed that resistance to LpxC inhibitors arises predominantly from mutations in *fabZ*, where reduced dehydratase activity facilitates *E. coli* survival under conditions of LpxC inhibition (68). These genetic interactions are consistent with substrate competition at the *R*-3-hydroxymyristoyl-ACP branchpoint: reduced FabZ activity increases substrate availability for lipid A synthesis, compensating for diminished LpxA or LpxC function. Conversely, hyperactive FabZ diverts more *R*-3-hydroxymyristoyl-ACP into fatty acid elongation and incorporation into phospholipids, limiting substrate availability under conditions of elevated LpxC. Together, these results support a model in which modulation of *R*-3-hydroxymyristoyl-ACP flux balances LPS and phospholipid synthesis.

Beyond substrate competition, multiple studies demonstrate that fatty acid synthesis enzymes, particularly FabZ, modulate LpxC stability. Gain-of-function *fabZ* mutations stabilize LpxC, leading to higher cellular LpxC abundance and deacetylase activity (31, 68), whereas hypomorphic *fabZ* mutations reduce LpxC levels, consequently lowering cellular activity (67, 68). Perturbations in other fatty acid enzymes also regulate LpxC cellular abundance. Specifically, LpxC is stabilized in temperature-sensitive strains JP1111 [*fabI*(Ts)] and CY288 [*fabF*, *fabB*(Ts)] (69). In contrast, overexpression of FabA, FabD, FabF, or the transcriptional regulator FadR was reported to accelerate LpxC degradation, although this effect was observed under an LpxC overexpression background and may not reflect physiological regulation (70). Researchers have attempted to explain these findings with a feedback model in which flux through the lipid A pathway modulates LpxC proteolysis: enhanced flux increases LPS intermediates, thereby promoting LpxC degradation, whereas reduced flux stabilizes LpxC. Although this framework accounts for many observations, it clearly does not explain all of the experimental results. For example, *E. coli* encodes two β-hydroxyacyl-ACP dehydratases, FabZ and FabA (71). Although both purified enzymes exhibit comparable activity toward *R*-3-hydroxymyristoyl-ACP *in vitro* (71), genetic suppressors that restore LPS homeostasis consistently map to *fabZ* rather than *fabA*. Moreover, increased FabZ activity stabilizes LpxC, whereas overexpression of FabA was reported to accelerate LpxC degradation. However, the effect of FabA overexpression should be interpreted with caution, as the study was conducted in an LpxC overexpression background (70). These distinctions indicate that FabZ has a unique role in coordinating phospholipid and LPS synthesis, beyond mere substrate competition.

**Figure 6:**
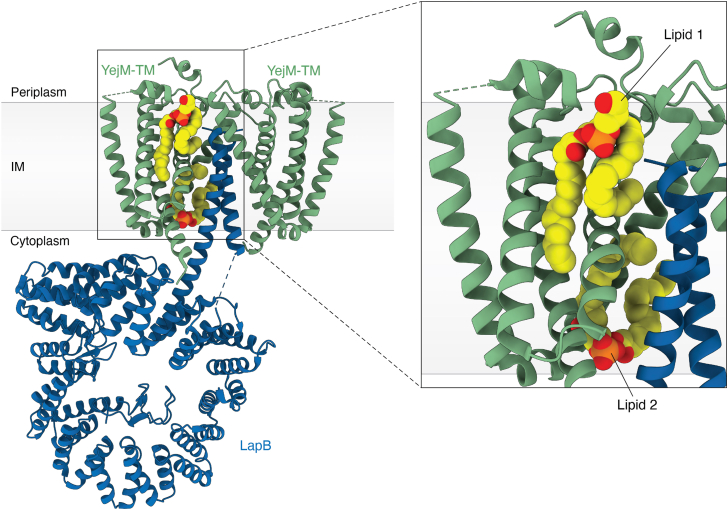
**Structure of the YejM/LapB complex.** Two phospholipid molecules (*yellow*) are at the interface. The *right panel* shows a magnified view of the boxed region in the *left panel*. (PDB-7T6D). IM, inner membrane; PDB, Protein Data Bank; YejM-TM, transmembrane domain of YejM.

Given that the substrate competition and simple feedback model of LPS synthesis cannot fully explain the effects of FabZ on LpxC, how might FabZ activity influence LpxC stability through additional mechanisms? New evidence suggests that the FtsH/LapB/YejM system may directly sense signals derived from fatty acid and phospholipid synthesis, in addition to LPS intermediates. For example, PlsY, a glycerol-3-phosphate acyltransferase involved in phospholipid synthesis, was identified in endogenous YejM immunoprecipitates (53), indicating a direct interaction between phospholipid synthesis enzymes and YejM in the regulation of LPS synthesis. Similarly, overexpression of AcpT, a holo-ACP synthase, suppresses the lethality of *yejM* null mutants (43, 44). Because catalytically inactive AcpT retains this suppressive effect (43), the rescue is unlikely to depend on its enzymatic role in lipid metabolism, but instead suggests a functional connection between fatty acid pathway components and the FtsH/LapB/YejM regulatory system. An intriguing possibility is that the phospholipid composition of the IM modulates the dynamic complex formation and dissociation in the FtsH/LapB/YejM pathway. The cryo-EM structure of YejM/LapB protein complex demonstrated that their interaction is mediated by phospholipids (Fig. 6), suggesting that the local membrane environment contributes directly to the regulation of LpxC degradation (54). In this framework, alterations in fatty acid synthesis could remodel IM phospholipid composition, thereby influencing YejM/LapB complex formation and ultimately LpxC turnover. This hypothesis provides a mechanistically plausible explanation for how phospholipid homeostasis might be coupled to regulated LpxC degradation and represents an interesting avenue for future investigation.

The regulatory relationship between phospholipid and LPS appears largely unidirectional: altering FabZ activity modulates LpxC abundance and thus its deacetylase activity, whereas reported LpxC mutations do not measurably affect cellular FabZ dehydratase activity (66, 67). This apparent asymmetry may reflect a metabolic hierarchy, in which the majority of fatty acids are incorporated into phospholipids, allowing phospholipid synthesis to set the overall pace of acyl flux while LPS synthesis adjusts accordingly. Consistent with this view, the Bokinsky laboratory showed that phospholipid synthesis adjusts according to cell growth rate, whereas LPS production is indirectly governed by *R*-3-hydroxymyristoyl-ACP availability (72). They further identified PlsB, the glycerol-3-phosphate acyltransferase that initiates phospholipid synthesis, as a key determinant of acyl flux partitioning, while LpxC levels remain relatively constant across cell growth rates (72). This observation contrasts with a previous report claiming that LpxC degradation rates vary with cell growth rate (73). Notably, those experiments were performed using overexpressed LpxC, whereas the Bokinsky study examined LpxC at endogenous levels. Together, these findings support a model in which phospholipid synthesis largely dictates LPS production in *E. coli*.

## Coordination of LPS and peptidoglycan synthesis: links to LpxC regulation

Beyond its well-established role as a permeability barrier, the OM is increasingly appreciated for its contributions to mechanical load-bearing and cell morphogenesis—processes traditionally attributed primarily to peptidoglycan (4, 5). Since the early elucidation of the LPS biosynthetic pathway, Raetz *et al*. recognized that LPS and peptidoglycan synthesis share a common precursor, UDP-GlcNAc (74). As with the balance between LPS and phospholipid synthesis, the partitioning of UDP-GlcNAc between LPS and peptidoglycan pathways must be tightly controlled. However, in contrast to the well-established coupling between FabZ and LpxC, relatively little is known about how LPS and peptidoglycan synthesis are coordinated beyond their shared use of this precursor in *E. coli*.

Recent studies from the Bernhardt laboratory provide the first evidence linking peptidoglycan synthesis to LpxC regulation in *E. coli* (5). Maintenance of rod shape of *E. coli* cells depends on the Rod complex (elongasome), a multiprotein machinery that coordinates lateral peptidoglycan synthesis during cell elongation. This complex is organized by the actin homolog MreB, which forms dynamic filaments along the IM and guides cell wall synthesis, and includes essential components such as MreC, MreD, RodA, and the transpeptidase PBP2 that together catalyze glycan polymerization and crosslinking. Perturbation of Rod complex function—either through treatment with the MreB inhibitor A22 or *via* hypomorphic mutations in MreC—leads to decreased LpxC abundance and reduced LPS levels. Whether this effect is mediated through the FtsH/LapB/YejM proteolytic pathway remains unclear. The morphological defects of *mreC* mutants can be partially rescued by increasing LpxC levels through mutations in *ftsH* or *lapB*, providing evidence for a functional contribution of LPS to cell morphogenesis. Again, the relationship appears to be unidirectional: reduced Rod complex activity lowers LpxC abundance, whereas elevated LpxC levels do not affect Rod complex activity. This asymmetry suggests that peptidoglycan synthesis may play a dominant role in dictating LPS production under these conditions.

Compared with the extensive body of work on the coordination between LPS and phospholipid synthesis, the mechanisms linking LPS and peptidoglycan biogenesis in *E. coli* remain poorly understood. Elucidating how these two essential biosynthetic pathways are integrated, represents an important area for future investigation.

## Control of LpxC enzymatic activity: an emerging paradigm

In general, metabolic flux can be regulated by modulating either enzyme abundance or enzyme activity. For LpxC, control of enzyme abundance in *E. coli* has dominated the field for decades, whereas direct regulation of LpxC deacetylase activity has only recently gained attention. In addition to its well-established role as an adaptor for FtsH-mediated LpxC degradation, LapB was recently shown to directly inhibit LpxC enzymatic activity *in vitro* (55), although whether this inhibition contributes to the regulation of LPS synthesis *in vivo* remains to be determined. This finding suggests that regulation of LpxC activity may operate alongside proteolytic control, providing an additional layer for tuning LPS synthesis.

Since both functions of LapB—as an adaptor that promotes LpxC degradation and as a direct inhibitor of LpxC enzymatic activity—ultimately reduce LPS levels, it is intriguing that *E. coli* has evolved what appears to be a redundant system. How these two functions are coordinated to regulate LPS synthesis remains unclear. Which function predominates under different physiological conditions, and how do their relative contributions vary with cell growth state, envelope stress, or metabolic status? In principle, inhibition of enzymatic activity could provide rapid, reversible control, whereas modulation of LpxC abundance *via* proteolysis may impose longer-term adjustments to pathway flux. Experimental strategies that separate LapB’s adaptor and inhibitor function, such as domain-specific mutations, will be critical to dissect how these mechanisms individually and collectively maintain LPS homeostasis.

Regulation of LpxC activity through protein–protein interactions is not limited to *E. coli*, nor is inhibition the only outcome. The Bernhardt group recently found that MurA in *Pseudomonas aeruginosa*, stimulates LpxC deacetylase activity—an effect not observed in *E. coli* (75). These observations indicate that modulation of LpxC enzymatic activity, whether stimulatory or inhibitory, represents a novel regulatory mechanism. This mechanism may reflect a broader, previously underappreciated strategy for coordinating LPS synthesis with envelope biogenesis.

The mechanism by which LapB inhibits LpxC deacetylase activity remains unknown, and elucidating it is an important goal given the therapeutic potential. LpxC has long been considered a promising antibacterial target, with most efforts focused on developing inhibitors that target its catalytic center (76, 77, 78). Representative inhibitors, such as the first high-affinity broad-spectrum inhibitor CHIR-090 (79), typically comprise a hydroxamate “warhead” that chelates the catalytic zinc ion, a hydrophobic moiety that fits into the tunnel formed by insertion II, thereby conferring specificity (76, 80). All these inhibitors compete with the substrate for the active site, thereby inhibiting LpxC competitively. Even compounds with exceptionally high affinity and potent activity against Gram-negative pathogens, such as LPC-233 that bind in the picomolar range (81), have not advanced to clinical use. In contrast, LapB does not compete with the substrate and instead inhibits LpxC allosterically. A deeper understanding of LpxC allosteric regulation could enable the development of next-generation inhibitors that target regulatory interfaces rather than the catalytic zinc-binding site, potentially yielding novel scaffolds with improved therapeutic potential.

## Concluding remarks

Over the past decades, substantial progress has been made in elucidating the regulation of LpxC and, more broadly, the mechanisms governing LPS biosynthesis and OM homeostasis in *E. coli*. Structural, genetic, and biochemical studies have defined the central roles of the FtsH/LapB/YejM pathway, substrate flux control, coordination among LPS, phospholipids and peptidoglycan synthesis, and emerging modes of enzymatic regulation. Nevertheless, several key questions remain regarding LpxC regulation: how signals from distinct cellular compartments are integrated, how FabZ and other metabolic enzymes affect LpxC stability beyond substrate competition, how peptidoglycan synthesis influences LpxC, how LpxC abundance and its enzymatic activity are regulated individually and together, and how its deacetylase activity is allosterically controlled. Resolving these issues will advance our understanding of LPS homeostasis and may uncover new therapeutic strategies against Gram-negative pathogens.

## Data availability

Not applicable.

## Conflict of interest

The authors declare that they have no conflicts of interest with the contents of this article.
